# Correction: iTRAQ-based quantitative proteomic analysis for identification of biomarkers associated with emodin against severe acute pancreatitis in rats

**DOI:** 10.1039/c9ra90061f

**Published:** 2019-08-23

**Authors:** Hong Xiang, Qingkai Zhang, Danqi Wang, Shilin Xia, Guijun Wang, Guixin Zhang, Hailong Chen, Yingjie Wu, Dong Shang

**Affiliations:** College (Institute) of Integrative Medicine, Dalian Medical University Dalian 116011 China shangdong@dmu.edu.cn +86-411-83622844 +86-411-83635963; Institute of Gene Engineered Animal Models for Human Diseases, Dalian Medical University Dalian 116044 China; Department of General Surgery, Pancreatico-Biliary Center, The First Affiliated Hospital of Dalian Medical University Dalian 116011 China; Clinical Laboratory of Integrative Medicine, The First Affiliated Hospital of Dalian Medical University Dalian 116011 China; Department of General Surgery, The First Affiliated Hospital of Jinzhou Medical University Jinzhou 121000 China

## Abstract

Correction for ‘iTRAQ-based quantitative proteomic analysis for identification of biomarkers associated with emodin against severe acute pancreatitis in rats’ by Hong Xiang *et al.*, *RSC Adv.*, 2016, **6**, 72447–72457.

The authors regret that Fig. 2–4 were shown incorrectly in the original article. An incorrect section of the SAP group in the MPO-immunohistochemical staining (Fig. 2A) and HE staining (Fig. 3) experiments was used in error. In addition, Fig. 4 has been revised to show the zymogen granule, in order to better represent the ultrastructure of the pancreas. The correct versions of Fig. 2–4 are shown below.

**Fig. 2:**
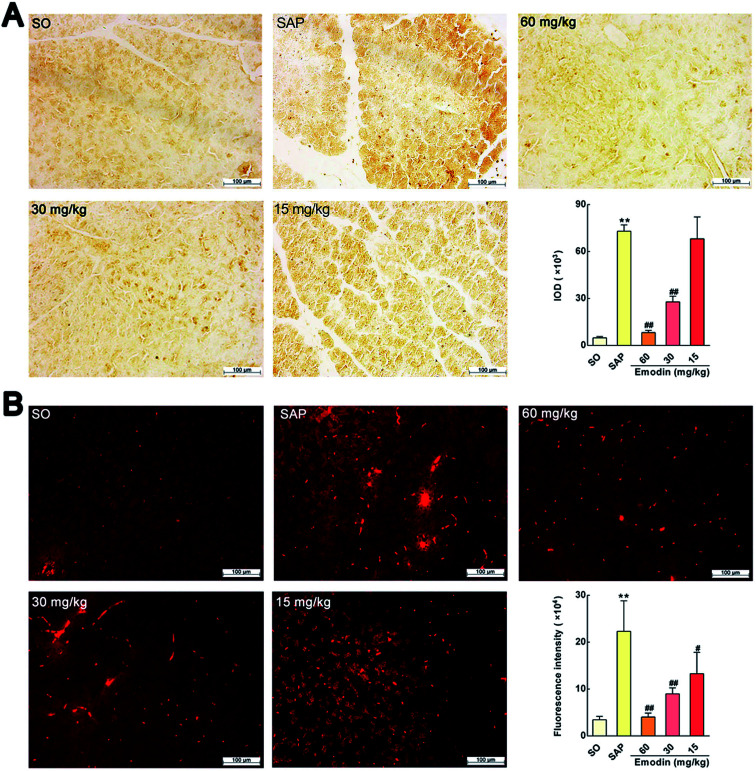
Emodin down-regulated the MPO protein expression in pancreas of SAP rats. (A) Effect of emodin on MPO-immunopositive area (brown) staining of pancreatic tissue in SAP rats by immunohistochemical detection. (B) Effect of emodin on MPO-immunopositive area (red) staining of pancreatic tissue in SAP rats by immunofluorescence detection. Images are presented at 200× magnification. The data are presented as the mean ± SD, *n* = 6. **P < 0.01 *versus* SO; ^#^*P* < 0.05 *versus* SAP, ^##^*P* < 0.01 *versus* SAP.

**Fig. 3:**
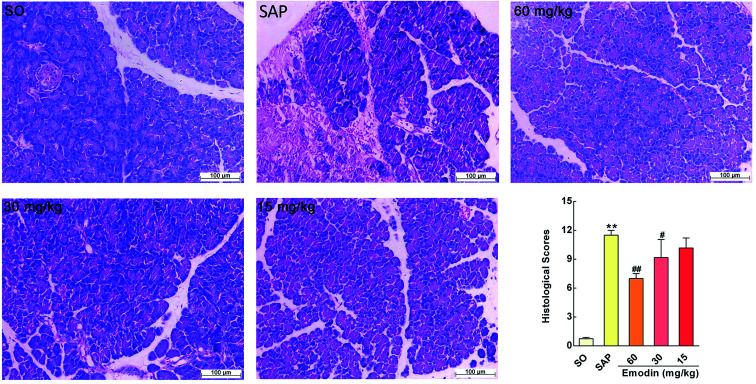
Emodin improved pancreatic histopathology of SAP rats. Effect of emodin on H&E staining of pancreatic tissue in SAP rats. Images are presented at 200× magnification. The data are presented as the mean ± SD, *n* = 6. **P < 0.01 *versus* SO; ^#^*P* < 0.05 *versus* SAP, ^##^*P* < 0.01 *versus* SAP.

**Fig. 4:**
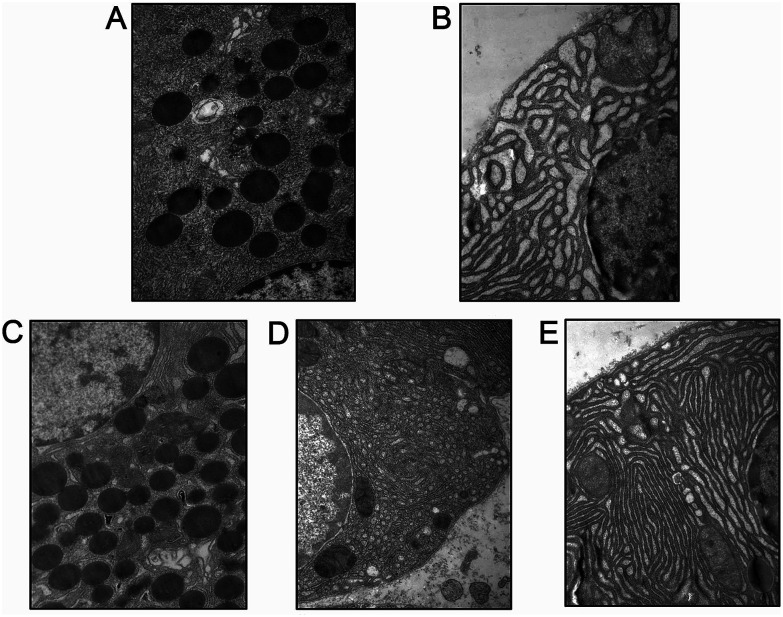
Emodin attenuated cellular structure changes in pancreas of SAP rats. Representative images of the cells’ ultrastructure in the SO (A), SAP (B), 60 mg kg^−1^ emodin (C), 30 mg kg^−1^ emodin (D) and 15 mg kg^−1^ emodin (E) groups. Images are presented at 25 000× magnification.

The Royal Society of Chemistry apologises for these errors and any consequent inconvenience to authors and readers.

## Supplementary Material

